# Investigation of Interactive Activity of Electro-Acupuncture on Pharmacokinetics of Sildenafil and Their Synergistic Effect on Penile Blood Flow in Rats

**DOI:** 10.3390/ijms19082153

**Published:** 2018-07-24

**Authors:** Andy C. Huang, Kuei-Ying Yeh, Yung-Yi Cheng, Navneet Kumar Dubey, Allen W. Chiu, Tung-Hu Tsai

**Affiliations:** 1 Institute of Traditional Medicine, School of Medicine, National Yang-Ming University, Taipei 11221, Taiwan; andyhuangmd@gmail.com (A.C.H.); vininecheng@gmail.com (Y.-Y.C.); whchiu@ym.edu.tw (A.W.C.); 2 Department of Urology, School of Medicine, National Yang-Ming University, Taipei 11221, Taiwan; 3 Department of Urology, Department of Surgery, Taipei City Hospital Ren-Ai Branch, Taipei 10629, Taiwan; 4 Department of Physical Therapy, College of Medicine and Health Care, HungKuang University, Taichung 43302, Taiwan; d93441003@ntu.edu.tw; 5 Ceramics and Biomaterials Research Group, Advanced Institute of Materials Science, Ton Duc Thang University, Ho Chi Minh City 700000, Vietnam; navneet.kumar.dubey@tdt.edu.vn; 6 Faculty of Applied Sciences, Ton Duc Thang University, Ho Chi Minh City 700000, Vietnam; 7 Graduate Institute of Acupuncture Science, China Medical University, Taichung 404, Taiwan; 8 School of Pharmacy, College of Pharmacy, Kaohsiung Medical University, Kaohsiung 807, Taiwan; 9 Department of Chemical Engineering, National United University, Miaoli 36063, Taiwan

**Keywords:** erectile dysfunction, acupuncture, electro-acupuncture, pharmacokinetics, sildenafil, penile blood flow

## Abstract

Erectile dysfunction (ED) is a disorder found in males throughout the world, which negatively affects relationships with partners with advancing age. Hence, in this study, we tested a combined novel treatment of electro-acupuncture (EA) and sildenafil citrate against ED. In addition to EA therapy, the sildenafil citrate, a phosphodiesterase 5 inhibitor, is a widely recognized drug that has achieved considerable success in the treatment of ED. However, the combined effect of both the EA and sildenafil has not yet been investigated. Hence, we aimed to examine the effect of EA on the pharmacokinetics and pharmacodynamics of sildenafil in rat plasma. The pharmacokinetic parameters were determined using ultra performance liquid chromatography (UPLC) after EA and sildenafil administration (10 mg/Kg). Following this, the pharmacodynamics was studied via blood flow pattern using developing Doppler images of the lower body and penis. The pharmacokinetic studies demonstrated that sildenafil significantly increases by administration of low-frequency EA. Further, the pharmacodynamic studies using Doppler imaging revealed an elevated blood flow in rat penis compared with lower body during combined treatment of sildenafil and low-frequency EA. These data indicate a synergistic therapeutic effect of EA and sildenafil for the treatment of ED.

## 1. Introduction

Erectile dysfunction (ED) is one of the major sexual problems throughout the world and has been reported to affect about 52% of males between the ages of 40 and 70 years [1,2]. The chances of ED increases with advancing age and negatively affects personal behavior and the relationship with a partner. Various therapeutic alternatives for treatment of ED, including injection of the vasoactive drug, oral drug therapy, psychosexual therapy, revascularization, penile prostheses, and vacuum constriction devices, have been employed [3]. These therapies are limited because of their potency and related side effects like penile pain, cardiovascular disease, and deafness [4,5,6]. Currently, the acupuncture in traditional Chinese medicine (TCM) is increasingly used to treat ED [7], because of its safety profile [8]. It has not only been acknowledged in controlling pain, but also in treating infectious and inflammatory, psychological, neurological, pulmonary, and cardiovascular disorders, among others. [2]. The World Health Organization also recognizes 29 diseases for which acupuncture therapy has been proven as an effective treatment [3]. In acupuncture, the two important acupoints, Guanyuan (CV4) and Zongji (C3), have demonstrated the potential benefits for ED [9]. During acupuncture, the sterile thin needles are inserted into the skin at these acupoints to stimulate the natural process of healing. Specifically, the acupuncture stimulates afferent nerve fibers such as A-β fiber, A-δ fiber, and C-fibers during treatment procedures. Recently, Lee et al. reported that sweet bee venom injection stimulates CV4, leading to an improvement of sexual dysfunction [10]. The effect of acupuncture can be further improvised by applying some external stimulation factors, such as electrical stimulus in the form of electro-acupuncture (EA), to stimulate acupoints via pulsed electric voltage instead of providing a stimulus similar to that obtained by a manual needle [11]. Therefore, EA may provide stronger and continuous stimulus to reach the enhanced efficacy of treatment compared with conventional acupuncture. Notably, the administration of acupuncture on blood circulation is also a considerable factor for determining treatment efficacy [7]. Further, the EA stimulations at both high (80–100 Hz) and low frequency (2–4 Hz) have been reported for their effectiveness in the treatment of various disorders [12,13,14,15]. Though the exact mechanism of EA is not well established, it was reported that at low frequency, electrical stimulation imparts tonic response during stimulation; whereas, at high frequency, the phasic response is generated during the initial stage of stimulation [16].

Besides, sildenafil is a widely accepted drug for treatment of ED. In a seminal study, Natali et al. reported that the sildenafil (50 mg) improved sexual performance by increasing the blood flow in cavernous arteries in patients with ED [17]. In another important study, the effect of *Epimedium sagittatum* (Sieb. et Zucc.) maxim extract on sildenafil pharmacokinetics was reported with significant herb–drug interaction. Based on this study, it was suggested that the herb enhances the elimination of the drug, and it needs to be carefully considered before using both sildenafil and herb together in the treatment of ED [18]. Therefore, we hypothesized that the combined administration of EA and sildenafil might be a potential therapeutic alternative for ED.

To our knowledge, this is the first study to examine the combined effect of EA and sildenafil, in which the pharmacokinetics of sildenafil via its interaction with EA in rats was assessed. Furthermore, their pharmacodynamics was studied by determining blood flow in the rat penis and lower body through Doppler image analysis. Taken together, this study may be helpful for developing a combinatorial therapy against ED by using EA and sildenafil.

## 2. Results

### 2.1. Analytical Method Validation for Sildenafil

The chromatograms of sildenafil are represented in Supplementary Figure S1A–C, which revealed retention time of the internal standard: p-hydroxybenzoate (IS: PHB) and sildenafil as 4.4 min and 9.8 min, respectively. The chromatogram of rat blank plasma was shown in Figure S1A, and the peak for blank plasma spiked with sildenafil (1 µg/mL) is represented in Figure S1B. The Figure S1C is characteristic chromatogram of rat plasma sample after 5 min of sildenafil administration. The formula, accuracy (%) = (C_obs_ − C_nom_/C_nom_) × 100, was used to calculate the accuracy. The mean nominal concentration and mean observed concentration were represented by C_obs_ and C_nom_, respectively, whereas precision was determined through relative standard deviation (RSD). For RSD calculation, the formula, RSD (%) = (standard deviation (SD)/C_obs_) × 100, was used. Data for determination of analytical precision and accuracy of rat plasma is shown in Supplementary Table S1. The calibration curve for sildenafil had good linearity (r^2^ = 0.995) over a range of 0.1 μg/mL to 10 μg/mL. The inter-day and intra-day assay had relative standard deviation values less than 15.90%, while the accuracy rate ranged from 9.26% to 15.4%. These results showed no significant difference between the initial and tested concentrations of sildenafil.

### 2.2. Effect of Sildenafil and EA on the Pharmacokinetic Parameters

**Figure 1:**
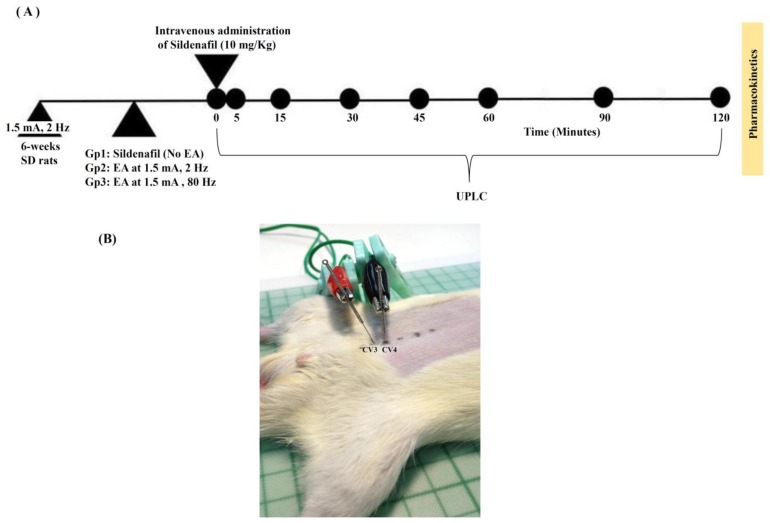
Pharmacokinetics experimental design (**A**). The study treatment group was divided into three groups. Group 1: sildenafil (10 mg/Kg, i.v.) treated only; group 2: sildenafil (10 mg/Kg, i.v.) + low-frequency electro-acupuncture (EA) (1.5 mA, 2 Hz) treated; and group 3: sildenafil (10 mg/Kg, i.v.) + high-frequency EA (1.5 mA, 80 Hz) treated for animal experiment (**B**). Blood sample was collected from the right external jugular vein at 0, 5, 15, 30, 45, 60, 90, and 120 min after sildenafil administration. GP: group; SD: Sprague-Dawley; UPLC: ultra performance liquid chromatography.

**Figure 2:**
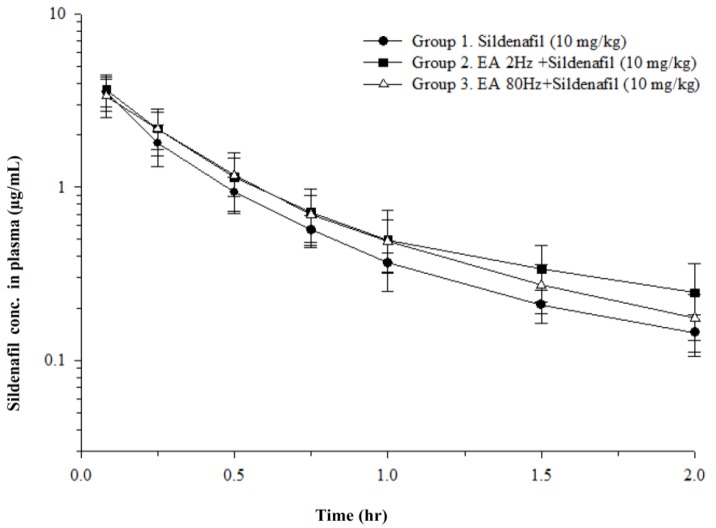
Plasma concentration-time profile of intravenous sildenafil in the blood of group 1—sildenafil (10 mg/kg) alone; group 2—sildenafil (10 mg/kg) + low frequency (2 Hz) EA; group 3—sildenafil (10 mg/kg) + high frequency (80 Hz) EA. Data expressed as mean ± S.D. (*n* = 6).

The schematic design of this study is illustrated in Figure 1 and the dosage regimens of sildenafil and EA administration for group 1, 2, and 3 are defined as sildenafil (10 mg/Kg), sildenafil (10 mg/Kg) + low-frequency EA (1.5 mA, 2 Hz), and sildenafil + high-frequency EA (1.5 mA, 80 Hz), respectively (Table 1). Pharmacokinetic parameters of rat plasma for sildenafil (10 mg/Kg), sildenafil (10 mg/Kg) + low-frequency EA (1.5 mA, 2 Hz), and sildenafil + high-frequency EA (1.5 mA, 80 Hz) treated groups are enlisted in Table 2. Figure 2 shows plasma concentration-time profile of sildenafil in the rat plasma of all groups. The results revealed slightly higher sildenafil concentration in rats with combined treatment of low- or high-frequency EA and sildenafil, as compared with the group treated with sildenafil only. Initially, there was no difference recorded in the concentration of sildenafil in group 2 and 3 for the first one hour. After one hour, a higher concentration of sildenafil was detected in rat plasma of group 2 as compared with group 3; however, it was found to be insignificant. Moreover, when compared with group 1, group 2 revealed significantly higher levels of sildenafil. Taken together, the pharmacokinetic data (Table 2) demonstrate that compared with high-frequency (80 Hz), the low-frequency EA (2 Hz) might elevate the concentration of sildenafil in the blood (*p* < 0.05).

### 2.3. Efficacy of Sildenafil and EA on Pharmacodynamics Parameters 

**Figure 3:**
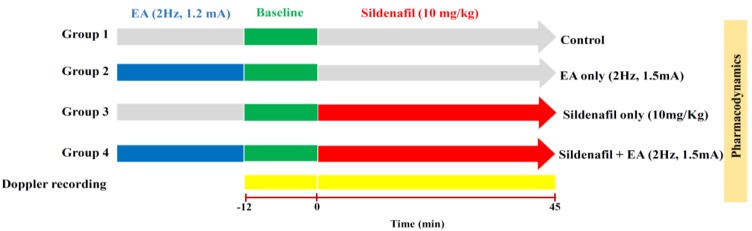
The schematic of pharmacodynamics study with Doppler imaging after sildenafil and EA administration. Rats were placed into four groups: group 1: control (no sildenafil or EA treatment), group 2: only EA treated, group 3: only sildenafil (10 mg/ Kg, i.v.) treated, and group 4: sildenafil (10 mg/Kg, i.v.) + EA (1.5 mA, 2 Hz) treated. The lower body and penis blood flow pattern was monitored for 45 min and images were captured for analysis after every 3 min. To determine control data for blood flow, the blood flow was monitored 12 min before treatment, and it was considered as the baseline.

**Figure 4:**
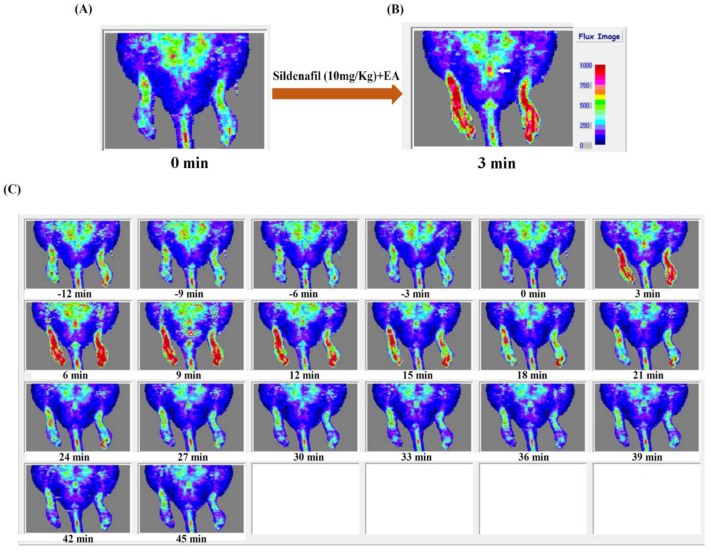
Doppler images of penile and lower body blood flow in an electro-acupunctured rat at 1.5 mA and 2 Hz. (**A**) The representative laser Doppler images show the blood flow prior to sildenafil administration; and (**B**) reveals the increase in blood flow in the lower body and penis (white arrow) after direct stimulation by combined treatment of sildenafil and EA at 3 min. (**C**) Continuous monitoring of blood flows in the lower body and penis until 45 min.

**Figure 5:**
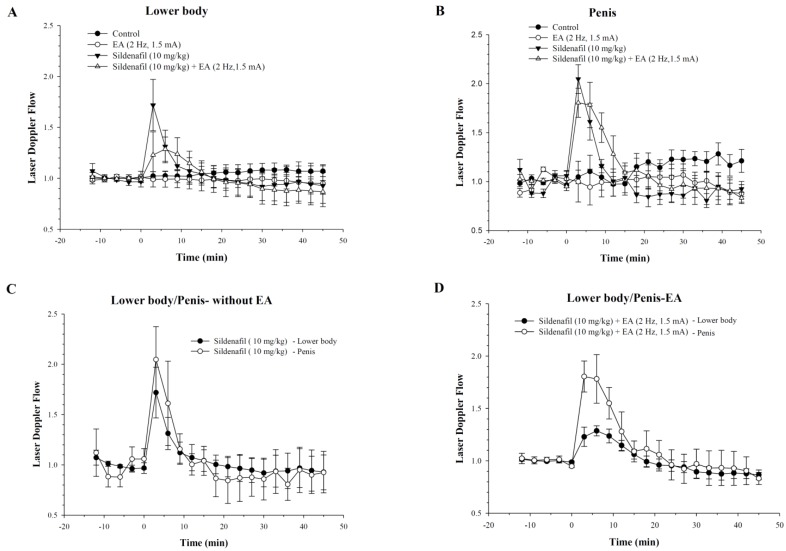
Blood flow and time profile in penis and lower body of rats after combined treatment of both EA (1.5 mA, 2 Hz) and sildenafil. After the stimulation, the blood flow changes were determined in the (**A**) lower body and (**B**) penis; and (**C**) a comparative lower body to penis without EA and (**D**) with EA. The *Y*-axis represents the ratio of the response level compared with pre-treatment value.

As the plasma concentration profile showed a higher concentration of sildenafil at lower frequency (1.5 mA, 2 Hz), we selected this frequency for pharmacodynamics studies using Doppler imaging to assess penile and lower body blood flow in rats after sildenafil and EA administration (Figure 3). Figure 4 depicts the penile and lower body blood flow in an electro-acupunctured rat at 1.5 mA and 2 Hz. In Figure 4A, the representative laser Doppler images show blood flow prior to sildenafil administration. While Figure 4B reveals the increased blood flow at 3 min after combined treatment of sildenafil and EA. The Doppler images of penile and lower body blood flow in rat are demonstrated in Figure 4C. After administration of both the sildenafil and EA, these images were captured every 3 min for consecutive 45 min. The baseline of 12 min (−12 to 0 min) before sildenafil administration was set for measurement of blood flow. The results revealed that the time to reach the highest blood flow was around 6 min. Statistical analysis demonstrated a significant increase in blood flow in the lower body and the penis of rats treated with sildenafil and EA, as compared with control groups, as well as those without EA treatment (*p* < 0.01). These results were further quantified as demonstrated in Figure 5A–D. When sildenafil was administered alone, the higher blood flow was observed in the penis compared with the lower body (Figure 5C). Interestingly, after combined treatment of both sildenafil and EA (1.5 mA, 2 Hz), a prolonged time of high blood flow in the penis (*p* < 0.01) was observed when compared with the lower body (Figure 5D), indicating concentrated blood supply in the penis.

## 3. Discussion

ED is a most common problem reported throughout the world. Many traditional therapeutic approaches are used to overcome this problem; however, the use of sildenafil citrate has demonstrated a considerable improvement in symptoms of ED. On the other hand, the EA is also one of the established traditional methods for the treatment of many disorders [12,19], though its role in the treatment of ED is yet to be determined. Besides, the impact of combined treatment of EA and sildenafil also remains to be evaluated. Thus, this study mainly focused on establishing the effect of EA on the pharmacokinetics of sildenafil for the treatment of ED in rats. As demonstrated in Table 2, the pharmacokinetics parameter, AUC of group 2 Sildenafil [(10 mg/Kg) + EA (1.5 mA, 2 Hz)] was higher than group 1 [Sildenafil (10 mg/Kg) only] (*p* = 0.026), whereas, no significant difference was observed between group 2 and group 3 [Sildenafil (10 mg/Kg) + EA (1.5 mA, 80 Hz)] (*p* = 0.59). Thus, the above finding delineates that low-frequency EA may increase sildenafil concentration in the rat body. This might be supported by the fact that low-frequency EA may impact sildenafil metabolism because sildenafil is known to be metabolized by liver microsomes and CYP3A4 [20]. In a previous study by Wu et al., no significant effect of acupuncture and EA on the pharmacokinetics of aspirin and salicylic acid was found [15,19,21,22]. Another study reported that EA treatment with a high dose of paracetamol significantly affected some of the pharmacokinetic parameters and needs to be further clinically evaluated to overcome any undesired side-effects of drug dose and EA treatment altogether. Similarly, EA also decreases the intra-operative remifentanil consumption and enhances the negative effects of sinusotomy [22,23]. However, Aloe and Manni were able to control hyperalgesia induced by a repeated dose of nerve growth factor (NGF) with low-frequency EA [24], indicating that EA could be helpful to control this kind of disorder. Some other studies have also been carried out to establish the interaction between herbal drugs and EA/acupuncture; however, the results were inconsistent according to the acupoints and drugs/herbs used [25,26].

There are several factors, including plasma protein binding, blood perfusion, membrane permeability, enzymatic metabolism, and membrane transports, that are known to influence the pharmacokinetics of a drug [27]. However, we conducted a pharmacodynamic study to establish the possible interaction between EA and sildenafil treatment. The penile and lower body blood flow in rat was monitored by developing Doppler color images, and baseline for blood flow measurement was considered 12 min before sildenafil administration. The increase in blood flow was represented in red (Figure 4). Penile and lower body blood flow in rat was continuously monitored for 45 min to measure the change in blood flow in control and experimental groups. In the Doppler experiments, sildenafil + EA treatment showed increasing blood flow, especially in rat penis. Notably, the increased blood flow period in the penis was also prolonged compared with other groups. Though the administration of EA reduces the maximum flow rate in the lower body and penis (Figure 5A,B), the blood flow rate in lower body and penis was decreased and increased, respectively, after combined therapy of sildenafil and EA (Figure 5C,D). This increased penile blood flow indicates the entry of a higher volume of blood into the penis. Therefore, it could be inferred that EA might improve the efficacy of sildenafil by increasing the concentration of blood into penis, while reducing the flow rate in the lower body. These results were clearly related to the EA treatment at low frequency. Thus, it could be pointed out that low-frequency EA has an interactive effect on sildenafil pharmacodynamics in rats. This also suggests that EA may act in synergy with sildenafil by not only increasing circulation, but also lengthening sildenafil effect during treatment of ED. However, the choice of acupoints in EA is a critical factor, and the impact of EA on the circulatory effect of sildenafil need to be determined carefully, as it maybe affect the absorption, distribution, metabolism, and excretion of the molecule of interest [28]. Interestingly, the combined treatment of sildenafil and low-frequency EA demonstrated an enhanced blood flow in the penis compared with the lower body (Figure 4), which implies the concentrated blood supply in sinusoidal spaces of the penis. According to previous reports, the vessel dilation governing penile erection relies on nitric oxide (NO), which is produced both in cavernosal nerves and endothelium of blood vessels. As sildenafil (one of the phosphodiesterase (PDE)-5 inhibitors) increases intracellular concentrations of cyclic guanosine monophosphate during NO signaling, it might lead to dilatation of vessels. Therefore, we assume that EA in synergism with sildenafil may enhance blood flow via stimulating cavernosal nerves to induce NO release [29,30,31]. Notably, in addition to various positive outcomes, our study also possesses a few limitations. As the time of administration between EA and sildenafil is very close, it is thus difficult to predict the exact time of the lasting effect of EA. It is also not possible to conduct sexual activity immediately after combined therapy of sildenafil and EA. Further, in our study, the EA treatment was done only once; hence, the effect of multiple stimulation of EA (e.g., once daily for 5 days) should also be investigated. Overall, further extensive studies are necessary to verify the effectiveness of this synergistic treatment.

## 4. Materials and Methods

### 4.1. Animals and Study Design

We used Sprague-Dawley (SD) rats of 6–7 weeks of age, weighing 250 ± 20 g. The rats were maintained in a defined and constant environmental condition (temp: 23 ± 2 °C, Humidity: 55 ± 10%). Food and drinking water were freely available to the rat cages. The experimental methods and procedures were approved (IACUC approval number 1030612; valid from 06/01/2014 to 12/30/2015) by the Animal Ethical Committee of the Laboratory Animal Center at National Yang-Ming University Taipei, Taiwan (approval number 1030612, 1 June 2014). The study treatment group was divided into three groups: only sildenafil (10 mg/Kg, i.v.) treated, sildenafil (10 mg/Kg, i.v.) with low-frequency EA (1.5 mA, 2 Hz) treated, and sildenafil (10 mg/Kg, i.v.) with high-frequency EA (1.5 mA, 80 Hz) treated for pharmacokinetic study. Before the experiments, vascular cannulation was performed for intravenous sildenafil injection at the left femoral vein, while another vascular cannulation was performed at the right external jugular vein for sequential blood sampling. A blood sample of 0.2 mL in size was collected from the right external jugular vein at 0, 5, 15, 30, 45, 60, 90, and 120 min following sildenafil administration. Whereas in the study of blood flow by Doppler imaging, rats were placed into four groups: control (no sildenafil or EA treatment), only EA treated, only sildenafil-treated, and sildenafil (10 mg/Kg, i.v.) + EA (1.5 mA, 2 Hz) treated. During the experiments, urethane (1 g/mL) and α-chloralose (0.1 g/mL) at a dosage of 1 mL/kg body weight of mice were intraperitoneally administered for anesthesia.

### 4.2. UPLC-UV Instrumentation

Sildenafil citrate was purchased from Pfizer Pharmaceuticals (New York, NY, USA) and p-hydroxybenzoate (PHB, Wako Pure Chemical Industries, Ltd., Osaka Japan) was purchased and used as standard and internal standard (IS), respectively. Our detection method followed the guidelines of USFDA and found no interaction between sildenafil and the internal standard. Thus, we considered that this internal standard is suitable, and it was used for further investigation. Liquid chromatographic grade solvent and reagents were purchased from E. Merck (Darmstadt, Germany). Triple deionized water was prepared by the Milli-Q system (Millipore, Bedford, MA, USA). The plasma sample was spiked with IS; blood sample was collected and immediately centrifuged for plasma separation. UPLC-UV instrumentation was performed with a Shimadzu chromatographic pump (LC-20AT), a DGU-20A5 degasser, an autosampler (SIL-20AC), and a photo-diode array detector (SPD-M20A, Shimadzu, Kyoto, Japan). Phenomenex Kinetex C18 column (100 × 2.10 mm, 2.6 μm, Torrance, CA, USA) was used. The mobile phase consists of acetonitrile/10 mM KH2PO4 + triethylamine (99.5:0.5, *v*/*v*, pH 6.0 adjusted with orthophosphoric acid) at the ratio 70:30 (*v*/*v*). Flow rate and the UV detection wavelength were set at 0.3 mL/min and 220 nm, respectively. The HPLC injected volume was 10 μL. The method was linear, selective, and precise for the range of 0.1 µg/mL to 10 µg/mL sildenafil. The protocol of plasma preparation was followed as described in Hsueh’s report [18]. Briefly, the liquid–liquid extraction was used for sildenafil extraction. The blood samples (100 μL) were extracted by ethyl acetate (500 μL), then vortex-mixed for 5 min, and centrifuged at 6000× *g* for 10 min. These steps were repeated thrice, thereafter, the ethyl acetate extraction solutions were mixed. After evaporating the combined extraction solution to dryness, the residues were reconstituted in 100 μL mobile phase. Before HPLC analysis, the samples were centrifuged at 3200× *g* for 10 min and filtered through 0.22 μm membrane filter.

### 4.3. Sildenafil and Electro-Acupuncture (EA) Administration

On the midline of the lower abdomen, CV4 (Guanyuan) and CV3 (Zhong Ji) are located at 3/5 and 4/5 of the way from the umbilicus to the upper edge of the pubic bone. Six SD rats were selected in each group, anesthetized, and put into ventral position and this region was shaved and cleaned with 70% ethanol. Sterile acupuncture needles (outer diameter of 0.28 mm) were inserted at two acupoints CV3 (Zhong Ji) and CV4 (Guanyuan). Needles were connected to electric pulse generator (positive electrode at CV3, negative electrode at CV4). EA treatment was delivered at 2 Hz or 80 Hz, 1.5 mA for 30 min, and then sildenafil (10 mg/Kg, i.v.) was administered via the femoral vein. 

### 4.4. Pharmacokinetics Parameters

The blood collected from rat and plasma was separated for determination of sildenafil concentration by UPLC-UV. Pharmacokinetic parameters: t_1/2_: elimination half-life; AUC: area under curve S; CL: clearance, Vss: the volume of distribution at steady state; MRT: mean residence time; were calculated by using WinNonlin Standard Edition Version 1.1 (Scientific Consulting Inc., Apex, NC, USA).

### 4.5. Measurement of Blood Flow in Lower Body and Rat Penis

For the lower body and penis, each group of rats were resolved under Doppler imager (moorLDI2, Moor Instruments, Millwey, UK). The blood flow pattern was monitored for 45 min and images were captured for analysis after every 3 min. To determine control data for blood flow, the blood flow was monitored 12 min before treatment, which was considered the baseline. The data obtained from Doppler was analyzed by using Moor LDI Version 5 Research Software (Moor Instruments, Devon, UK).

### 4.6. Statistical Analysis

Data were recorded as the mean ± standard deviation (S.D.). Statistical tool SPSS 10.07 was used for one-way analysis of variance (ANOVA) and post hoc analysis tests were carried to compare the data of each study groups. When the *p*-value was less than 0.05, the difference was considered statistically significant. The pharmacokinetic parameters were calculated by WinNonlin (version 1.1; Scientific Consulting Inc., Apex, NC, USA).

## 5. Conclusions

In our study, the pharmacokinetic results demonstrated that low-frequency EA might elevate sildenafil (10 mg/kg, i.v.) concentration in rat plasma, while pharmacodynamic investigation revealed that combined treatment of sildenafil and low-frequency EA enhanced rats’ penile circulation. Therefore, we inferred that the combined therapy of sildenafil and low-frequency EA might be a good candidate for the treatment of ED.

## Figures and Tables

**Table 1:** The dosage regimens of sildenafil and electro-acupuncture (EA) administration: group 1—sildenafil (10 mg/Kg) only; group 2—sildenafil (10 mg/Kg) + low frequency electro-acupuncture (1.5 mA, 2 Hz); group 3—sildenafil + high frequency electro-acupuncture (1.5 mA, 80 Hz).

Treatment Groups	Sildenafil (10 mg/Kg) and Electro-Acupuncture (mA, Hz) Dose
Group 1	Sildenafil (10 mg/Kg) only
Group 2	Sildenafil (10 mg/Kg) + EA (1.5 mA, 2 Hz)
Group 3	Sildenafil (10 mg/Kg) + EA (1.5 mA, 80 Hz)

**Table 2:** Pharmacokinetics parameters of sildenafil in various groups.

Parameters	C_0_ (μg/mL)	t_1/2_ (min)	AUC (min μg/mL)	Cl (mL/min/kg)	V_ss_ (mL/kg)	MRT (min)
Group 1	5.0 ± 1.0	43 ± 22	110 ± 15	92 ± 11	3866 ± 2450	41 ± 23
Group 2	4.8 ± 0.8	52 ± 27	137 ± 52	86 ± 44	3544 ± 831	50 ± 22
Group 3	4.3 ± 1.3	43 ± 10	124 ± 27	84 ± 20	3544 ± 787	43 ± 6

Group 1: sildenafil (10 mg/Kg) only; group 2: sildenafil (10 mg/Kg) + low frequency electro-acupuncture (1.5 mA, 2 Hz); Group 3: sildenafil + high frequency electro-acupuncture (1.5 mA, 80 Hz). Data represented as mean ± S.D. t_1/2_: elimination half-life; AUC: area under curve S; CL: clearance, Vss: the volume of distribution at steady state; MRT: mean residence time; EA: electro-acupuncture. Group 2 demonstrated significantly higher levels of sildenafil compared with group 1 (*p* < 0.05).

## Supplementary Materials

The following are available online at http://www.mdpi.com/1422-0067/19/8/2153/s1, Figure S1: UPLC chromatograms of intravenous sildenafil after administration of electro-acupuncture (EA). (A) blank plasma, (B) blank plasma spiked with sildenafil (1 μg/mL), (C) Plasma sample after sildenafil administration (10 mg/Kg, i.v.) + low frequency EA (1.5 mA, 2 Hz) administration. IS, Internal Standard (p-hydroxybenzoate); S, sildenafil. Table S1: Precision (% R.S.D.) and accuracy (% Bias) data for sildenafil.
