# Supplementary material for: Investigation of Interactive Activity of Electro-Acupuncture on Pharmacokinetics of Sildenafil and Their Synergistic Effect on Penile Blood Flow in Rats
Source: Int J Mol Sci. 2018 Jul 24;19(8):2153. doi: 10.3390/ijms19082153 (PMC6121473; doi:10.3390/ijms19082153)
Supplement: Supplementary file 1 [file ijms-19-02153-s001.pdf]

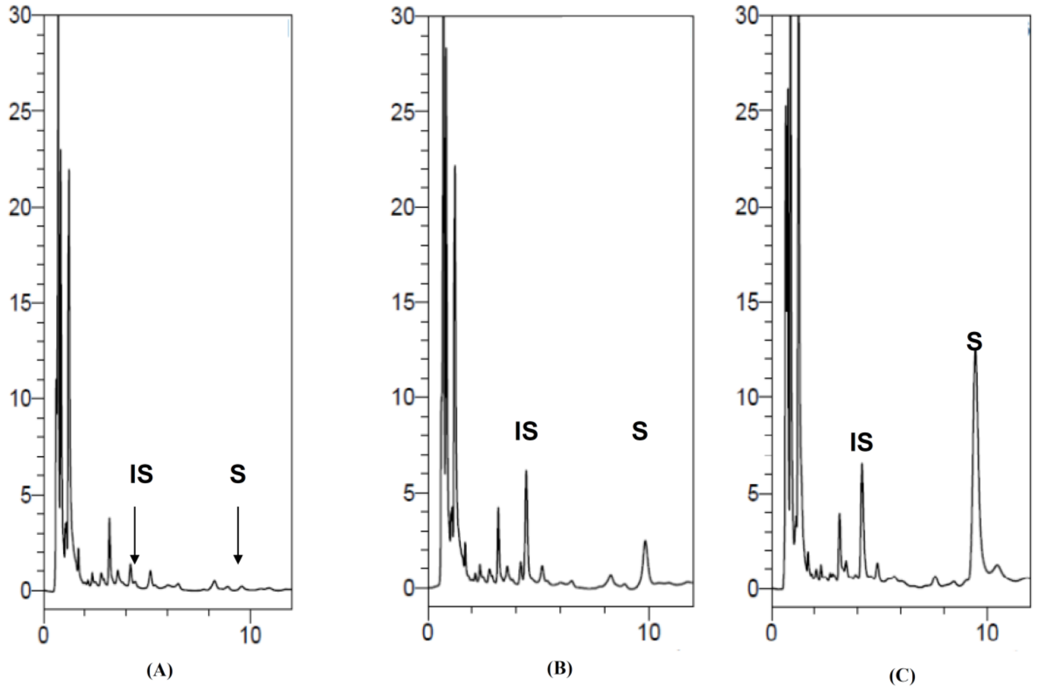

Figure S1. UPLC chromatograms of intravenous sildenafil after administration of electro-acupuncture (EA). (A) blank plasma, (B) blank plasma spiked with sildenafil (1 μg/mL), (C) Plasma sample after sildenafil administration (10mg/Kg, i.v.) + low frequency EA (1.5 mA, 2 Hz) administration. IS, Internal Standard (*p*-hydroxybenzoate); S, sildenafil.

Table S1. Precision (%R.S.D.) and accuracy (% Bias) data for sildenafil.

Nominal concentration (μg/mL)	Observed concentration (μg/mL)	RSD (%)	Accuracy (%)
Inter-day			
0.1	0.12 ± 0.02	15.9	15.4
0.5	0.50 ± 0.02	4.81	0.54
1	0.97 ± 0.02	2.38	3.29
5	4.93 ± 0.10	1.98	1.44
10	10.02 ± 0.05	0.52	0.24
Intra-day			
0.1	0.12 ± 0.02	14.6	19.2
0.5	0.45 ± 0.01	2.98	9.26
1	0.94 ± 0.02	2.27	5.78
5	5.15 ± 0.07	1.40	3.08
10	9.93 ± 0.03	0.34	9.69

Data are expressed as mean ± S.D., (n = 6). The relative standard deviation (RSD) was calculated from the observed concentration as follows: RSD (%) = [Standard deviation (S.D) / Observed concentration (C_{obs})] × 100. The accuracy was estimated by using mean value of nominal concentration (C_{nom}) and observed concentration (Cobs) as following: Accuracy (%) = [(C_{nom} - C_{obs})/C_{nom}] × 100 %.
